# Supplementary material for: Effects of Trichoderma harzianum combined with Phanerochaete chrysosporium on lignin degradation and humification during chicken manure and rice husk composting
Source: Front Microbiol. 2025 Feb 28;16:1515931. doi: 10.3389/fmicb.2025.1515931 (PMC11906335; doi:10.3389/fmicb.2025.1515931)
Supplement: Supplementary file 1 [file Data_Sheet_1.zip › 6.Function/All/FunGuild/l2/PLSDA-score.FunGuild_l2.All.pdf]

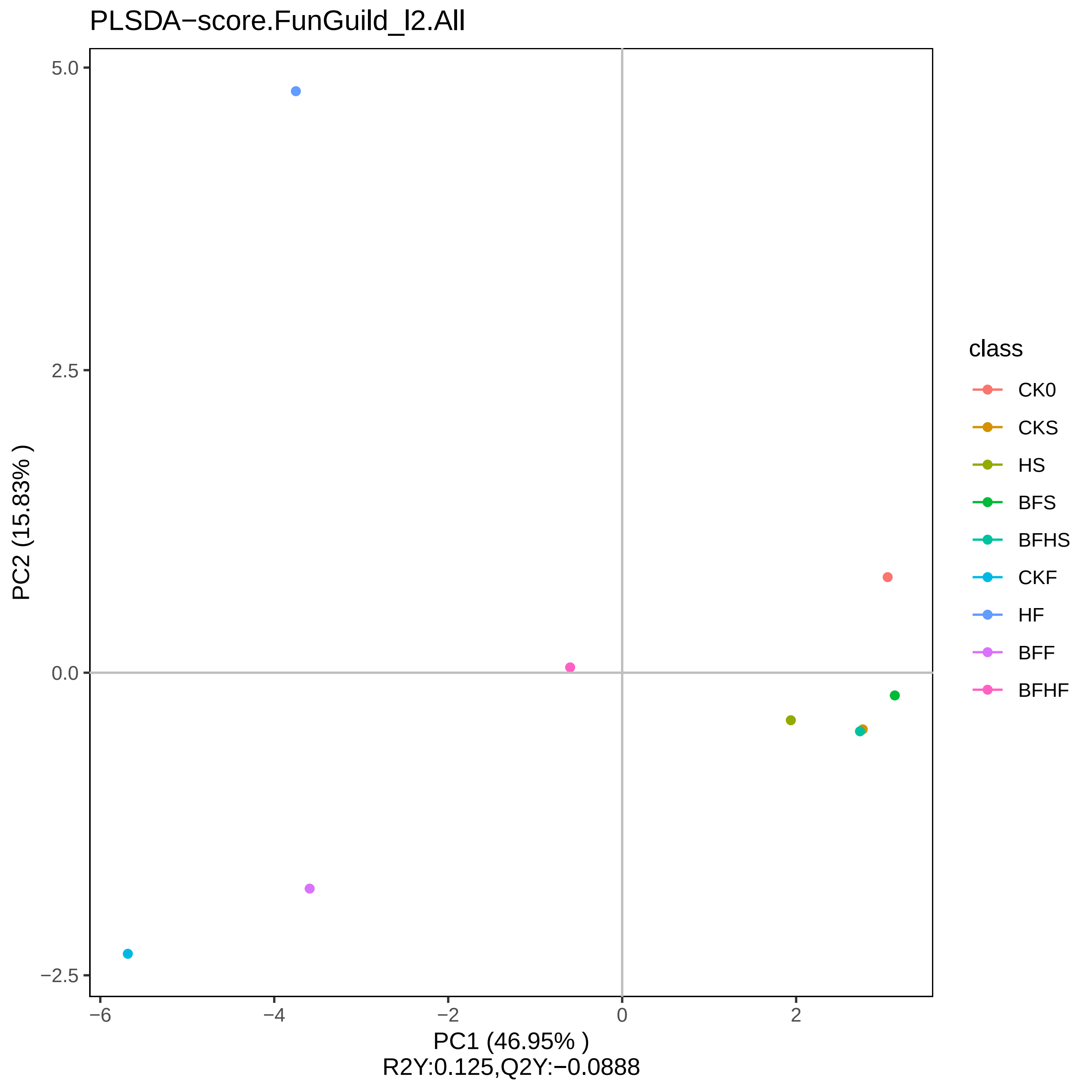

PLSDA−score.FunGuild_l2.All
5.0
2.5
0.0
−2.5
PC2 (15.83%)
−6
−4
−2
0
2
PC1 (46.95%)
R2Y:0.125,Q2Y:−0.0888
class
CK0
CKS
HS
BFS
BFHS
CKF
HF
BFF
BFHF
